# Multimodal Pain Recognition in Postoperative Patients: Machine Learning Approach

**DOI:** 10.2196/67969

**Published:** 2025-01-27

**Authors:** Ajan Subramanian, Rui Cao, Emad Kasaeyan Naeini, Seyed Amir Hossein Aqajari, Thomas D Hughes, Michael-David Calderon, Kai Zheng, Nikil Dutt, Pasi Liljeberg, Sanna Salanterä, Ariana M Nelson, Amir M Rahmani

**Affiliations:** 1 Department of Computer Science University of California, Irvine Irvine, CA United States; 2 Department of Electrical Engineering and Computer Science University of California, Irvine Irvine, CA United States; 3 School of Nursing University of California, Irvine Irvine, CA United States; 4 College of Medicine Kansas City University Kansas City, MO United States; 5 Department of Informatics University of California, Irvine Irvine, CA United States; 6 Department of Computing University of Turku Turku Finland; 7 Department of Nursing Science University of Turku Turku Finland; 8 Turku University Hospital University of Turku Turku Finland; 9 Department of Anesthesiology and Pain Medicine University of California, Irvine Irvine, CA United States; 10 Institute for Future Health University of California, Irvine Irvine, CA United States

**Keywords:** pain intensity recognition, multimodal information fusion, signal processing, weak supervision, health care, pain intensity, pain recognition, machine learning approach, acute pain, pain assessment, behavioral pain, pain measurement, pain monitoring, multimodal machine learning–based framework, machine learning–based framework, electrocardiogram, electromyogram, electrodermal activity, self-reported pain level, clinical pain management

## Abstract

**Background:**

Acute pain management is critical in postoperative care, especially in vulnerable patient populations that may be unable to self-report pain levels effectively. Current methods of pain assessment often rely on subjective patient reports or behavioral pain observation tools, which can lead to inconsistencies in pain management. Multimodal pain assessment, integrating physiological and behavioral data, presents an opportunity to create more objective and accurate pain measurement systems. However, most previous work has focused on healthy subjects in controlled environments, with limited attention to real-world postoperative pain scenarios. This gap necessitates the development of robust, multimodal approaches capable of addressing the unique challenges associated with assessing pain in clinical settings, where factors like motion artifacts, imbalanced label distribution, and sparse data further complicate pain monitoring.

**Objective:**

This study aimed to develop and evaluate a multimodal machine learning–based framework for the objective assessment of pain in postoperative patients in real clinical settings using biosignals such as electrocardiogram, electromyogram, electrodermal activity, and respiration rate (RR) signals.

**Methods:**

The iHurt study was conducted on 25 postoperative patients at the University of California, Irvine Medical Center. The study captured multimodal biosignals during light physical activities, with concurrent self-reported pain levels using the Numerical Rating Scale. Data preprocessing involved noise filtering, feature extraction, and combining handcrafted and automatic features through convolutional and long-short-term memory autoencoders. Machine learning classifiers, including support vector machine, random forest, adaptive boosting, and k-nearest neighbors, were trained using weak supervision and minority oversampling to handle sparse and imbalanced pain labels. Pain levels were categorized into baseline and 3 levels of pain intensity (1-3).

**Results:**

The multimodal pain recognition models achieved an average balanced accuracy of over 80% across the different pain levels. RR models consistently outperformed other single modalities, particularly for lower pain intensities, while facial muscle activity (electromyogram) was most effective for distinguishing higher pain intensities. Although single-modality models, especially RR, generally provided higher performance compared to multimodal approaches, our multimodal framework still delivered results that surpassed most previous works in terms of overall accuracy.

**Conclusions:**

This study presents a novel, multimodal machine learning framework for objective pain recognition in postoperative patients. The results highlight the potential of integrating multiple biosignal modalities for more accurate pain assessment, with particular value in real-world clinical settings.

## Introduction

Pain is defined by the International Association for the Study of Pain as “an unpleasant sensory and emotional experience associated with actual or potential tissue damage or described in terms of such damage” [1]. Pain is a unique phenomenon that individuals experience and perceive independently. Younger et al [2] stated that pain is a subjective experience for which there is no current objective measure. Pain may be classified as either acute or chronic; Kent et al [3] described acute pain as encompassing the immediate, time-limited bodily response to a noxious stimulus that triggers actions to avoid or mitigate ongoing injury. Chronic pain was first defined loosely by Bonica [4] as pain that extends beyond an expected timeframe; currently, chronic pain is defined as “persistent or recurrent pain lasting longer than three months” [5]. The focus of this paper is on acute pain.

Acute pain is a common experience in the postanesthesia care unit in the immediate period following surgery. According to Chou et al [6], pain occurs in 80% of patients following surgery, and 75% of patients with pain report their pain as either moderate, severe, or extreme. Current guidelines for the assessment of pain in the postanesthesia care unit recommend using a Numerical Rating Scale (NRS) or Verbal Rating Scale for patients who are sufficiently awake and coherent to reliably report pain scores [7]. However, Herr et al [8] identified several patient populations who are at risk for being incapable of providing self-report scores of pain; specifically, these populations include the pediatric population who have yet to develop adequate cognition; older patients with dementia; individuals with intellectual disabilities; and those who are unconscious, critically ill, or terminally ill. In these patient populations, Small and Laycock [7] recommend the use of behavioral pain scales, such as the Pain Assessment in Advanced Dementia, Critical Care Pain Observation Tool (CPOT), or Behavioral Pain Scale. Despite the pain assessment measures of self-report and behavioral pain scales, each of these methods may be prone to biases. For example, Craig et al [9] discussed how self-reporting might be a means to obtain a particular goal that can be influenced by the individual reporting pain. In addition, Hadjistavropoulos and Craig [10] provided the Communications Model of Pain, which provided a basis for how expressive behaviors are decoded by observers of individuals in pain, which are influenced by the message clarity transmitted by the individual in pain as well as the unique biases (eg, knowledge level, assessment skills, and predisposing beliefs) of the individual assessing pain. The difficult nature of interpreting pain scores has resulted in disparities in pain management in minority populations, with research by Staton et al [11] showing that the Black race is a significant predictor of the underestimation of pain by physicians.

Multimodal pain assessment represents a potential method of circumventing the limitations of traditional self-report and behavioral pain assessment tools and an opportunity for enhancing pain assessment in vulnerable populations. Instead of having to rely on only one dimension of pain assessment, such as behaviors through the use of the CPOT or Behavioral Pain Scale, future multimodal pain assessment will incorporate physiological indicators, such as electrodermal activity (EDA), electrocardiogram (ECG), electroencephalogram, and electromyogram (EMG) as well as behaviors (eg, facial expression), and perhaps other as-yet undiscovered parameters to capture pain assessment in patient populations that might not be best represented by current assessment strategies. For example, a study by Gélinas et al [12] found that revisions to the CPOT were necessary because some brain-injured patients may not exhibit certain behaviors that are contained in the CPOT. Similarly, for individuals diagnosed with dementia, Achterberg et al [13] stated that there is a preponderance of observer-based pain assessment tools, however, these tools retain significant differences between them, as well as concerns for lack of reliability, validity, and sensitivity of change. Enhancing pain assessment through the combination of traditional pain assessment methods with novel multimodal approaches may serve to eventually enhance pain assessment in a greater majority of vulnerable patient populations.

With the advent of connected Internet of Things devices and wearable sensor technology, automated data collection may achieve continuous pain intensity measurement. A significant amount of research has been conducted in recent years, which has sought to develop methods of continuous, automatic, and multimodal pain assessment. For example, previous work conducted by Walter et al [14] and Werner et al [15] used skin conductance level, ECG, electroencephalogram, and EMG to monitor pain in response to thermal pain. Other works, such as Hammal and Cohn [16] and Werner et al [17], have incorporated facial expression monitoring as an indicator of pain. While these studies were immensely beneficial to the scientific community in terms of their contributions to a better understanding of techniques to obtain continuous pain assessment, the setting of these experiments was in highly controlled laboratory environments with healthy participants. Collecting data in real-world situations as opposed to a laboratory setting would allow the researchers to assess a pain assessment technique’s potential in relation to actual pain brought about through a surgical procedure instead of induced pain.

The aim of this study is to develop a robust and effective multimodal pain assessment framework for postoperative patients in real clinical settings. To the best of our knowledge, this is the first work proposing a multimodal pain assessment framework for postoperative patients. It should be noted that a pain assessment study on real patients is associated with several challenges (eg, imbalanced label distribution, missing data, motion artifacts, etc) since several parameters such as the intensity, distribution, frequency, and time of the pain as well as the environment cannot be controlled by researchers. Our main contributions are 4-fold:

1. We conducted a clinical study for multimodal signal acquisition from an acute pain unit of the University of California, Irvine Medical Center.
2. We propose a multimodal pain assessment framework using our database (iHurt Pain DB) collected from postoperative patients while obtaining a higher accuracy compared to existing works on healthy participants [17].
3. We use both handcrafted and automatically generated features outputted from deep learning networks to build our models.
4. We provide a novel method to mitigate the presence of sparse and imbalanced labels (due to the real clinical setting of the study) using weak supervision and minority oversampling.

## Methods

### Overview

Candidates were selected from the Acute Pain Service patient list at University of California Irvine Health in Orange, California. The Acute Pain Service unit at the medical center serves approximately 100 patients weekly, enabling the lead Doctor of Medicine to recruit patients. This is the first claimed study that collected biosignals from postoperative adult patients in hospitals. All participants (aged 23-89 years) were recruited to the study from July 2018 to October 2019.

### iHurt Study Design

We conducted a biomedical data collection study on 25 postoperative patients reporting various degrees of pain symptoms. Multimodal biosignals (ECG, EMG, EDA, and photoplethysmography [PPG]) were collected from patients likely having mild to moderate pain who were asked to perform a few light physical activities while acquiring data. We also collected primary demographic information from each patient, including height, weight, sex, and BMI. All signals were collected using the iHurt system.

### iHurt System

iHurt is a system that measures facial muscle activity (ie, changes in facial expression) in conjunction with physiological signals such as heart rate, heart rate variability, respiration rate (RR), and EDA for the purpose of developing an algorithm for pain assessment in hospitalized patients. The system uses the following 2 components to capture raw signals.

#### Eight-Channel Biopotential Acquisition Device

Our team at the University of Turku, Finland, developed a biopotential acquisition device to measure ECG and EMG signals. The device incorporates commercially available electrodes, electrode-to-device lead wires, an ADS1299-based portable device, and computer software (LabVIEW version 14.02f, National Instruments) to visualize data streaming from the portable device. Raw signals from the electrodes are sampled at 500 samples per second and are sent to the computer software through Bluetooth for visualization [18].

#### Empatica E4

We use the commercially available Empatica E4 wristband (Empatica Inc) [19] to measure EDA and PPG signals. The purpose of using a wristband was to allow our participants to move freely without any impediments. The Empatica E4 was connected to the participants’ phones over Bluetooth for visualization.

We removed 3 participants’ data from the final dataset due to the presence of excessive motion artifacts. We also excluded 2 additional patients since they were wearing the Empatica E4 watch on their arm that received intravenous medication. This resulted in unreliable EDA signals due to conditions like skin rash and itching. This left us with data from 20 patients to build our pain recognition system. The dataset also contains rich annotation with self-reported pain scores based on the 11-point NRS from 0 to 10. A detailed explanation of the dataset and the study design can be found in Kasaeyan Naeini et al [20]. We intend to make the deidentified dataset available to the research community for further analysis and applications.

### Data Processing Pipeline

**Figure 1:**
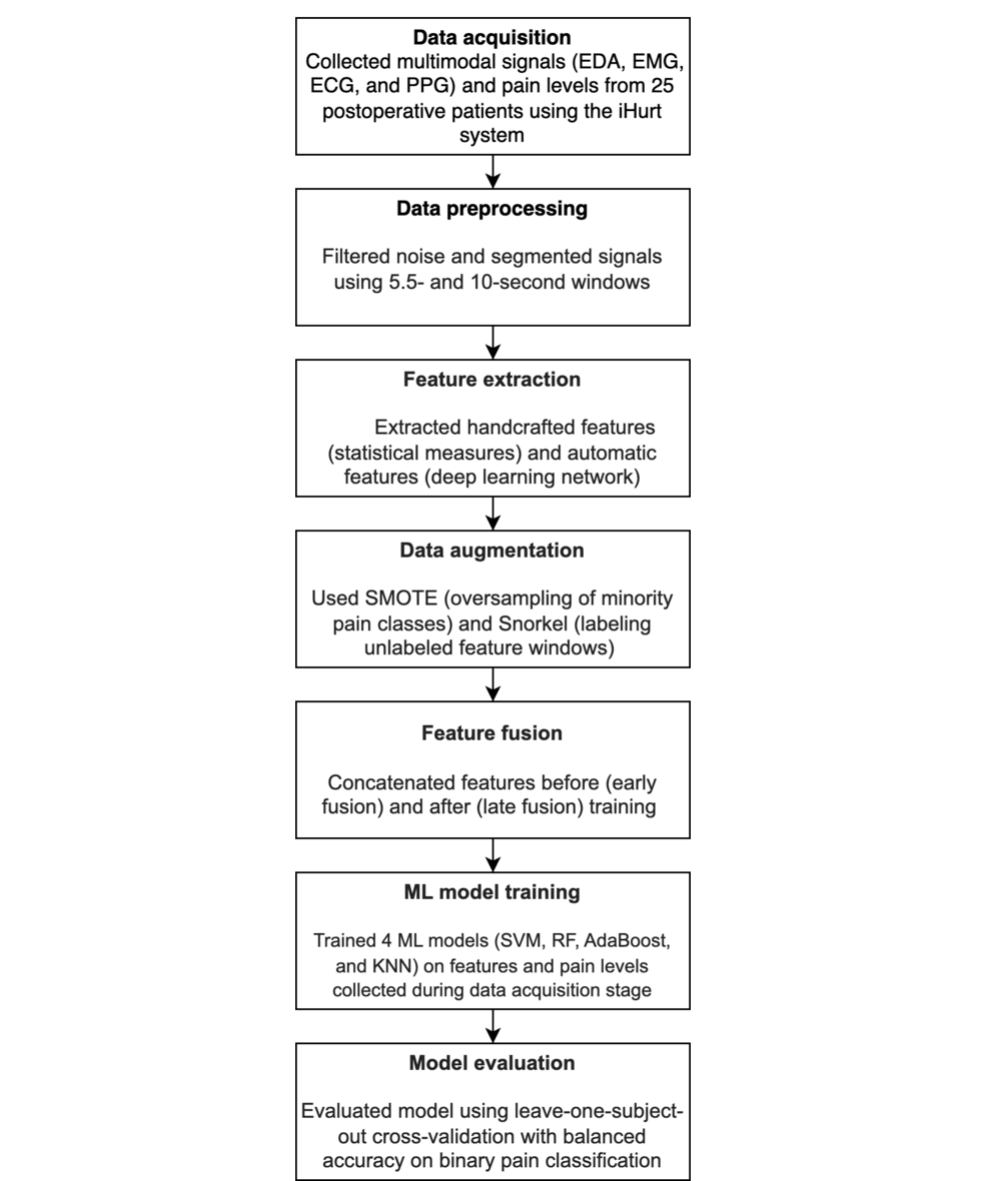
Overview of the proposed method. AdaBoost: adaptive boosting; EDA: electrodermal activity; EMG: electromyogram; ECG: electrocardiogram; ML: machine learning; PPG: photoplethysmography; SMOTE: synthetic minority oversampling technique; SVM: support vector machine; RF: random forest; KNN: k-nearest neighbors.

The first step in building our multimodal pain assessment system was to process the raw signals collected during trials. The data processing pipeline consisted of the following steps:

1. We filtered the signal to remove powerline interference, baseline wander, and motion artifact noise.
2. We performed feature extraction on the filtered signals to obtain amplitude and variability features in the time domain. The time domain features were extracted using 5.5-second and 10-second windows. The 5.5-second window size was extracted to be compared with previous work [17].
3. In addition to handcrafted features, we also used automatic features, which were outputted from a deep neural network.
4. Once the features were extracted, we tagged them with their corresponding labels based on the nearest timestamp of the label.
5. Each of these processing steps was applied individually to each of the 4 modalities. Processed data from each of the modalities were combined using either early fusion or late fusion. The types of handcrafted features extracted from each modality and the deep learning pipeline for extracting automatic features are described in detail. An overview of our method is described as a flowchart in Figure 1.

### ECG Handcrafted Features

The ECG channel was filtered using a Butterworth band-pass filter with a frequency range of 0.1-250 Hz. The heart rate variability handcrafted features were extracted with *pyHRV*, an open-source Python (Python Software Foundation) toolbox [21], using the R-peaks extracted from the ECG signal through a bidirectional long short-term memory (LSTM) network [22]. These features were extracted from two window sizes, 5.5 and 10 seconds. There were 19 time-domain features. The time-domain features extracted from NN intervals, or the time interval between successive R-peaks, comprised of the slope of these intervals, 5 statistical features (total count, mean, minimum, maximum, and SD), 9 difference features (mean difference, minimum difference, maximum difference, SD of successive interval differences, root mean square of successive interval differences, number of interval differences greater than 20 ms and 50 ms, and percentage of successive interval differences that differ by more than 20 ms and 50 ms), and 4 heart rate features (mean, minimum, maximum, and SD) [23].

### EMG Handcrafted Features

The preprocessing phase of EMG channels comprised a 20 Hz high pass filter and two notch filters at 50 Hz and 100 Hz, all using a Butterworth filter. Like ECG features, we extracted EMG features from 5.5- and 10-second windows on 5 different channels for each major facial muscle. The ten amplitude features extracted were (1) peak, (2) peak-to-peak mean value, (3) root mean squared, (4) mean of the absolute values of the second differences, (5) mean of the absolute values of the first differences, (6) mean of the absolute values of the second differences of the normalized signal, (7) mean of the absolute values of the first differences of the normalized signal, (8) mean of local minima values, (9) mean of local maxima values, and (10) mean of absolute values. The four variability features were (1) variance, (2) SD, (3) range, and (4) IQR. All 14 features were calculated for 5 different EMG channels, resulting in 70 EMG features in total.

### EDA Handcrafted Features

We used the *pyEDA* library [24] for preprocessing and feature extraction of EDA signals. In the preprocessing part, first, we used a moving average across a 1-second window to remove the motion artifacts and smooth the data [25]. Second, a low-pass Butterworth filter on the phasic data was applied to remove the line noise. Finally, preprocessed EDA signals corresponding to each different pain level were visualized to ensure the validity of the signals. In the feature extraction part, the *cvxEDA* algorithm [26] was used to extract the phasic component of EDA signals. The EDA signals’ peaks or bursts are considered variations in the phasic component of the signal. Therefore, the clean signals and extracted phasic component of signals were fed to the statistical feature extraction module to extract the number of peaks, the average value, and the maximum and minimum value of the signals. Furthermore, these extracted features were further used in the post–feature extraction module to extract eight more features: (1) the difference between the maximum and the minimum value of the signal, (2) the SD, (3) the difference between the upper and lower quartiles (4) root mean square, (5) the mean value of local minima, (6) the mean value of local maxima, (7) the mean of the absolute values of the first differences, and (8) the mean of the absolute values of the second differences. This resulted in 12 EDA features in total.

### PPG-Based RR Handcrafted Features

**Figure 2:**
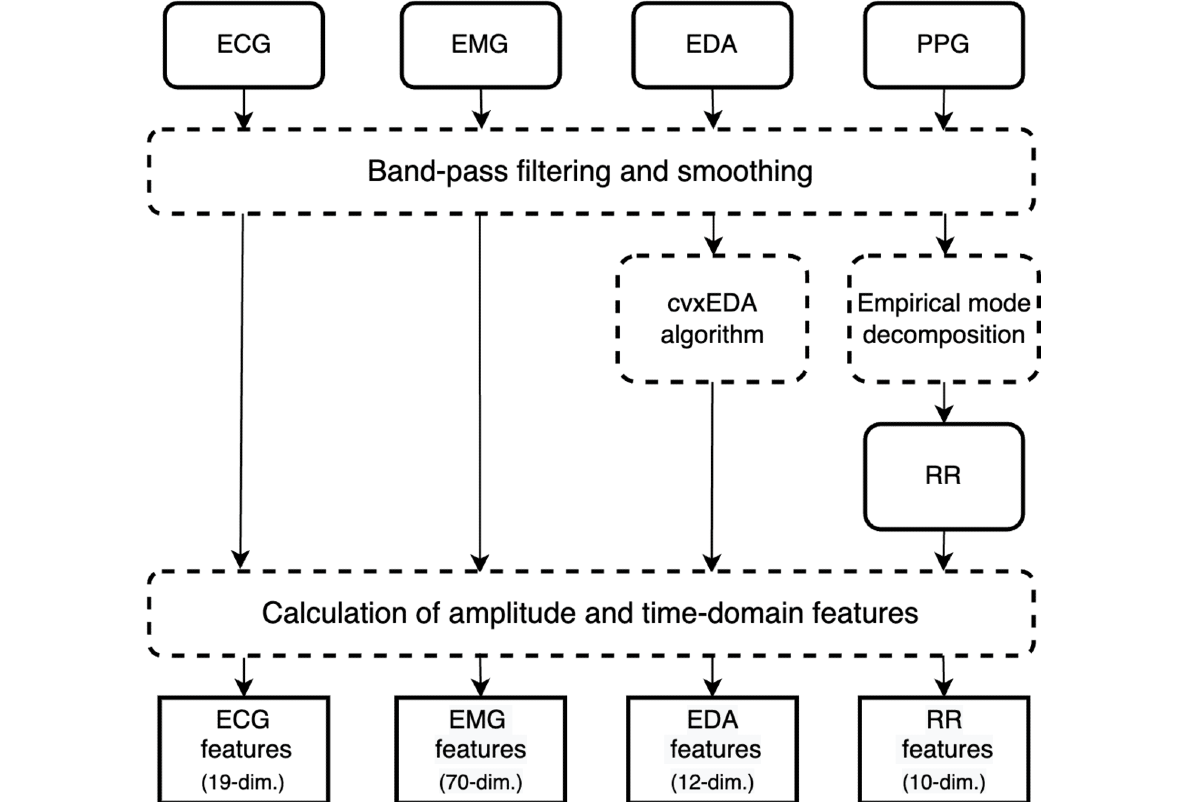
Handcrafted feature extraction pipeline. dim.: dimension; ECG: electrocardiogram; EDA: electrodermal activity; EMG: electromyogram; PPG: photoplethysmography; RR: respiration rate.

We preprocessed the PPG signal before extracting the RR from it. In total, 2 filters were used during the preprocessing [27]. We first used a Butterworth band-pass filter to remove noises, including motion artifacts. Then, a moving average filter was implemented to smooth the PPG signal. After that, we applied an empirical mode decomposition–based method proposed by Madhav et al [28] to derive respiration signals from filtered PPG signals. This method was proven to derive RR from a PPG signal with high accuracy (99.87%). A total of ten features were extracted from the respiratory signal, including (1) the number of inhale peaks, (2) the mean value of the signal, (3) the maximum value, (4) the minimum value, (5) the difference between the maximum and the minimum value, (6) SD, (7) the average value of the inhale peak intervals, (8) the SD of the inhale peak intervals, (9) the root mean square of successive differences between adjacent inhale peak intervals, (10) SD of inhale duration. A visualization of the handcrafted feature pipeline is shown in Figure 2.

### Automatic Feature Extraction Pipeline

As the dimensionality of biomedical data increases, it becomes increasingly difficult to train a machine learning algorithm on the entire uncompressed dataset. This often leads to a large training time and is computationally more expensive overall. A possible solution is to perform feature engineering to get a compressed and interpretable representation of the signal. Another alternative approach, however, is to use the compressed or latent representation of that data obtained from deep learning networks trained for that specific task. Using automatic features helps in dimensionality reduction and can provide us with a sophisticated yet succinct representation of the data that handcrafted features alone cannot provide. This automatic feature extraction is typically carried out by an autoencoder (AE) network, which is an unsupervised neural network that learns how to efficiently compress and encode the data into a lower-dimensional space [29,30]. AEs are composed of 2 separate networks: an encoder and a decoder. The encoder network acts as a bottleneck layer and maps the input into a lower-dimensional feature space. The decoder network tries to reconstruct this lower-dimensional feature vector into the original input size. The entire network is trained to minimize the reconstruction loss (ie, mean-squared error) by iteratively updating its weights and biases through backpropagation.

**Figure 3:**
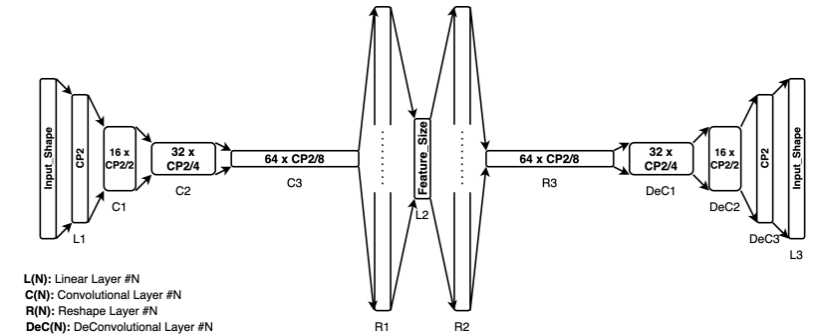
The architecture of the pyEDA convolutional autoencoder.

A convolutional AE from the *pyEDA* library was used to extract automatic features. Figure 3 shows the architecture of the AE. First, a linear layer (L1) is used to downsample the input signal with *Input_Shape* length to a length that is the closest power of 2 (CP2). This was done to make the model scalable to an arbitrary input size. The encoder half of the network consists of three 1D convolutional layers (C1, C2, and C3) and a linear layer (L2), which flattens and downsamples the input vector to a lower-dimensional latent vector. The number of dimensions of this latent vector (Feature Size) corresponds to the number of automatic features extracted and was set prior to training the network. A total of 32 features were extracted from ECG, EDA, and RR signals, whereas a total of 30 features were extracted from the EMG signal (6 features from each of the 5 channels). The decoder half of the network consists of three 1D deconvolutional layers (DeC1, DeC2, and DeC3) to reconstruct the input signal from the latent vector. A final linear layer (L3) is then used to flatten and reconstruct the signal to its original dimension. Both encoder and decoder networks have rectified linear unit activation between layers. Window sizes of both 5.5 and 10 seconds were applied to the filtered signals. This was done to compare the performance with handcrafted features. After signals from each of the modalities were normalized, they were trained on separate AE models for each modality. In addition to the convolutional AE, we also extracted features from an LSTM AE network. This resulted in two different feature extraction methods (convolutional and LSTM) that spanned two different window lengths (5.5 and 10 seconds).

**Figure 4:**
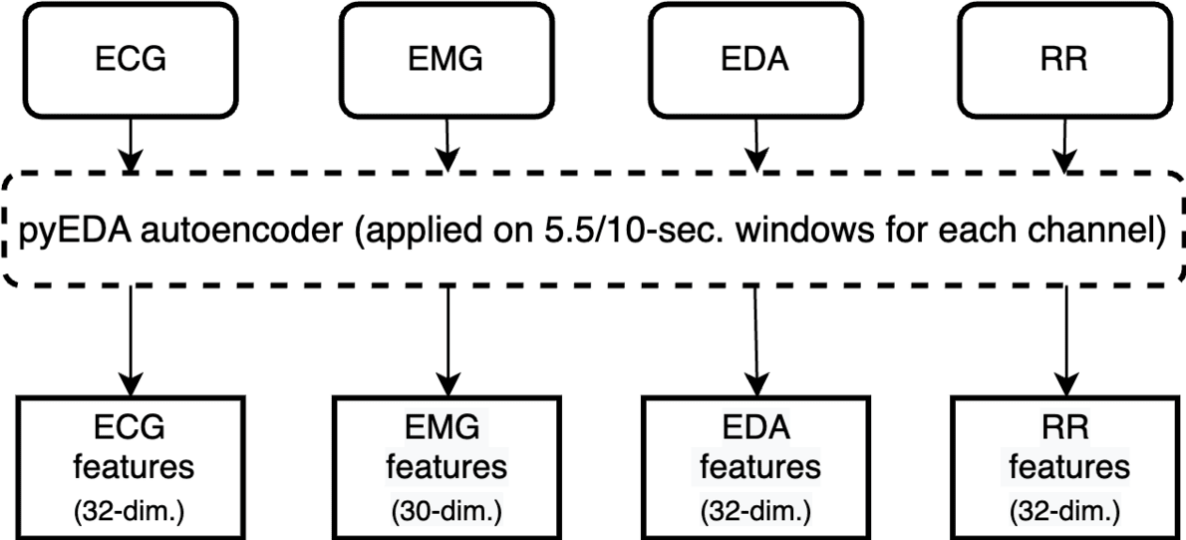
Automatic feature extraction pipeline. dim.: dimension; ECG: electrocardiogram; EDA: electrodermal activity; EMG: electromyogram; RR: respiration rate; sec.: second.

The batch size was set to 10, the number of training epochs was set to 100, and the ADAM optimizer [28] was used with a learning rate of 1 × 10^–3^. A total of 126 feature vectors across all 4 modalities were extracted from each AE network. A visualization of our automatic feature extraction pipeline is shown in Figure 4.

### Data Augmentation

#### Overview

There were several inherent challenges in the distribution of labels, as NRS values recorded during the clinical trials of this study were collected from real postoperative patients. This problem bears less significance while studying healthy participants since the stimulated pain can be controlled during the experiments. Consequently, occurrences of some pain levels far exceeded those of others. For example, among all patients, there were only 4 reported occurrences of pain level 10, whereas there were more than 80 reported occurrences of pain level 4. This imbalanced distribution was inevitable due to the subjective nature and the different sources of pain among the participants. Therefore, while downsampling our pain labels to 4 classes, thresholds for each downsampled class were carefully chosen to ensure a more evenly distributed set of labels. The pain levels ranged from a baseline (BL) level of pain or no pain to 3 increasing intensities of pain (PL 1-3). The thresholds for the pain levels were as follows: (1) PL1 ranged from 0 to 3, (2) PL2 ranged from 4 to 6, and (3) PL3 ranged from 7 to 10. All the ranges here are inclusive.

Since we asked patients to report their pain levels only while they performed pain-inducing activities, the number of labels generated was sparse. Both handcrafted and automatic features were combined with the corresponding labels using timestamps that were within the nearest 5.5 or 10 seconds (labeling threshold) of the reported NRS value. This depended on the window size of the features extracted. Due to having sparse labels, many of the feature windows were not assigned a corresponding label. To mitigate the problem of having an imbalanced and sparse label distribution, 2 techniques were exploited:

#### Minority Oversampling

The first technique, called synthetic minority oversampling technique (SMOTE), is a type of data augmentation that oversamples the minority class [31]. SMOTE works by first choosing a minority class instance at random and finding its k-nearest minority class neighbors. It then creates a synthetic example at a randomly selected point between two instances of the minority class in that feature space. The experiments involving SMOTE were implemented using the *imbalanced-learn* Python library [32].

#### Weak Supervision

The second technique we used is weak supervision using the Snorkel framework [33]. Rather than employing an expert to manually label the unlabeled instances, Snorkel allows its users to write labeling functions that can make use of heuristics, patterns, external knowledge bases, and third-party machine learning models. Weak supervision is typically employed to label large volumes of unlabeled data when there are noisy, limited, or imprecise sources. For our pain assessment algorithm, we decided to use third-party machine learning models to label the remaining unlabeled instances. All the data points that were within the labeling threshold were considered as “strong labels,” or ground-truth values collected from patients during trials. The remaining unlabeled data points were kept aside for Snorkel to provide a weakly supervised label. The strong labels were fed into Snorkel’s labeling function consisting of 3 off-the-shelf machine learning models: (1) a support-vector machine (SVM) with a radial basis function kernel, (2) a random forest (RF) classifier, and (3) a k-nearest neighbor (KNN) classifier with uniform weights. Once each model was trained on the strong labels, it was used to make predictions on the remaining unlabeled data. The predictions from these 3 models were collected and converted into a single confidence-weighted label per data point using Snorkel’s *LabelModel* function. This function outputs the most confident prediction as the label for each data point. To perform a fair assessment of the reliability and accuracy of our algorithm, we used SMOTE and Snorkel only while training our machine learning models. The performance of these models was measured solely on ground-truth (strong) labels collected during trials. This way, there is no implicit bias introduced from mislabeling or upsampling certain data points to skew model predictions.

### Multimodal Machine Learning Models

To compare the performance of our multimodal machine learning models with the previous work, we performed binary classification using a leave-one-subject-out cross-validation approach [34]. In this method, a model’s performance is validated over multiple folds in such a way that data from each patient are either in the training set or in the testing set. The purpose of using this method is to provide generalizability to unseen patients and to avoid overfitting by averaging the results over multiple folds. The eventual goal of this study is to build personalized models that make predictions on a single patient but learn from data collected from a larger population of similar patients. The following machine learning models were used to evaluate the performance of our pain assessment algorithm: (1) KNN, (2) RF classifier, (3) adaptive boosting (AdaBoost), (4) and an SVM. The models were then evaluated using leave-one subject-out cross-validation. Four separate models were trained for each of the 3 pain intensities (eg, BL; no pain versus PL1, the lowest pain level; or BL vs PL3, the highest pain level).

### Fusing Modalities

In total, 2 fusion approaches were used while combining features across different modalities. The first one is early or feature-level fusion, which concatenates feature vectors across different modalities based on their time stamps. The resulting data, which are now higher in dimension than any single modality, are then fed into our classifier to make predictions. While concatenating features across different modalities, a threshold of either 5.5 or 10 seconds was used to combine the modalities depending on the features extracted. The second approach was late or decision-level fusion, where each modality is fed to a separate classifier, and the final classification result is based on the fusion of outputs from the different modalities [35].

### Feature Selection

**Figure 5:**
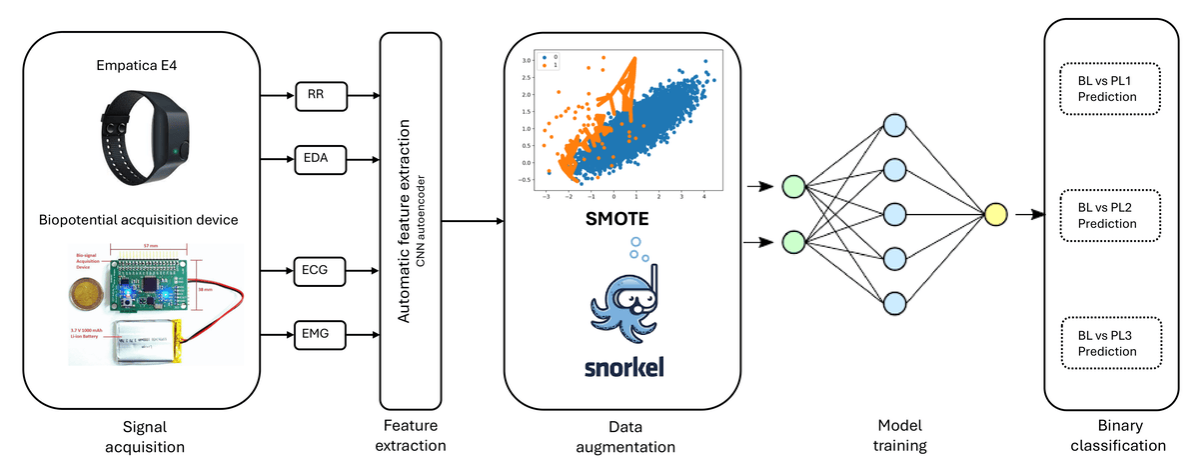
Proposed multimodal pain recognition system. BL: baseline; CNN: convolutional neural network; ECG: electrocardiogram; EDA: electrodermal activity; EMG: electromyogram; PL: pain level; RR: respiration rate; SMOTE: synthetic minority oversampling technique.

Since there were a lot of features generated during the data processing phase, we had to select a subset of the most informative features to build our models with. Therefore, to reduce the complexity and training time of the resulting model, feature selection using Gini importance was performed. Gini is important as a lightweight method that is simple and fast to compute. Since we extracted a relatively large number of features in our method, it made sense to use a computationally low-cost algorithm for feature selection. We computed the Gini importance of the features from the data in the training fold with the help of a random forest classifier and selected the top 25 features. We then trained our model on these top 25 features and evaluated them in the validation fold. Our proposed multimodal pain recognition system is shown in Figure 5.

### Ethical Considerations

The dataset used in this study was originally collected with approval from the Institutional Review Board (IRB) at the University of California, Irvine (Protocol HS# 2017-3747). Participants provided written informed consent after receiving detailed oral and written explanations of the study’s objectives and procedures. They were encouraged to discuss participation with family and friends before consenting. Investigators ensured that all participants understood the study and had their questions answered prior to enrollment. Participants were informed of their right to withdraw at any time without impacting their care. For the secondary analysis conducted in this study, the IRB approval and original informed consent covered the reuse of the data, and no additional consent was required. All data utilized for this study were anonymized prior to analysis to protect participants’ identities. Personal identifiers, such as names and contact information, were removed, and access to the data was restricted to authorized personnel only. The anonymized data were stored securely in compliance with institutional and regulatory guidelines to ensure confidentiality. Participation in the original study was entirely voluntary, and no compensation was provided. This ensured that participants’ involvement was based solely on their willingness to contribute to the research.

## Results

### Experimental Settings

**Figure 6:**
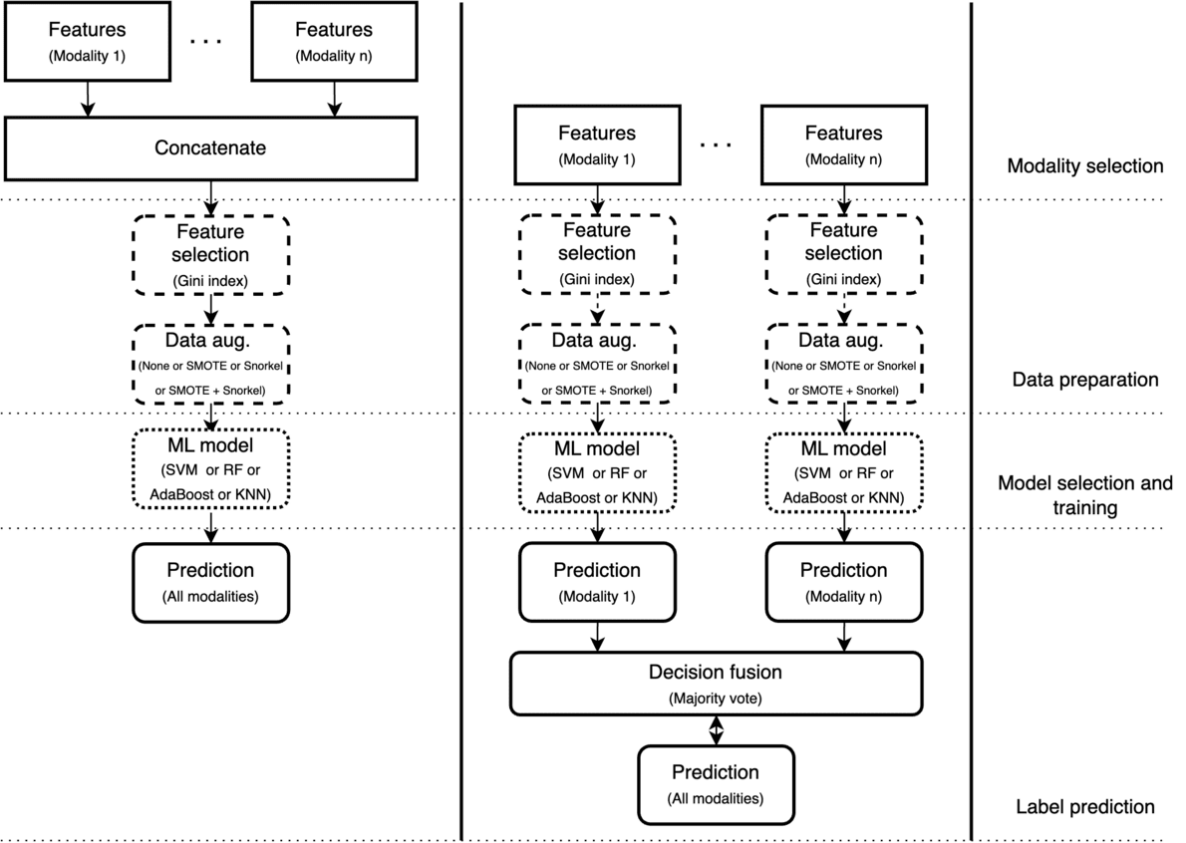
Our proposed general multimodal pipeline-based on early fusion (left) and late fusion (right). AdaBoost: adaptive boosting; KNN: k-nearest neighbors; ML: machine learning; RF: random forest; SVM: support vector machine; SMOTE: synthetic minority oversampling technique.

The goal of our experiments was to compare the performance of using only a single modality to build our models over using a combination of multiple modalities. We trained several different models for each of the pain intensities, which varied in the types of modalities, data augmentation techniques, machine learning models, and fusion techniques used. Figure 6 shows the general pipeline of the experiments we conducted. We first selected the type of modalities to train on, which varied from only using each of the single modalities separately to using a combination of all 4 modalities. Furthermore, these modalities varied depending on the type of features used, like handcrafted or automatic features. In the case of using multiple modalities, we had 2 choices of fusion: early (Figure 6, left) and late (Figure 6, right). These architectures varied in how the modalities were combined, either before training (early) or at the decision level (late) after training using majority voting. The data preparation process involved feature selection and data augmentation. These models could either be trained with no data augmentation, with just SMOTE or Snorkel, or a combination of both. The last step of the pipeline before making predictions involved choosing the type of machine learning algorithms, like SVM, RF, AdaBoost, or KNN. Due to the lack of space, only the best-performing single and multimodal model configurations are mentioned in the section below.

### Experimental Results

Tables 1 and 2 present the best-performing single-modal and multimodal models for each of the 3 pain intensities. For comparison, the best multimodal results from Werner et al [17], Lopez-Martinez and Picard [36], Wang et al [37], and Subramaniam and Dass [38] are also mentioned. We use balanced accuracy as an evaluation criterion because our dataset had an imbalanced class distribution. Balanced accuracy is defined as the average of the true positive rate and the true negative rate.

**Table 1:** Best scores: single modality versus multiple modalities.

Pain levels	ECG^a^ scores	EMG^b^ scores	EDA^c^ scores	RR^d^ scores	Multiple modality
BL^e^ vs PL^f^1	82.14	86	79.18	84.62	82.14
BL vs PL2	86.11	84.53	82.94	88.24	86.11
BL vs PL3	75	78.12	75	76.23	75
Mean (SD)	81.08 (5.03)	82.8 (5.03)	79.04 (5.03)	83.03 (5.03)	81.08 (4.59)
Classifier configuration	LSTM^g^ AE^h^ (10 s), Strong, SVM^j^	HC^i^ (10 s), Snorkel, SVM	CNN^k^ AE (10 s), Strong, SVM	HC (10 s), Strong, SVM	EF^l^, LSTM AE (10 s), Strong, SVM

^a^ECG: electrocardiogram.

^b^EMG: electromyogram.

^c^EDA: electrodermal activity.

^d^RR: respiration rate.

^e^BL: baseline.

^f^PL: pain level.

^g^LSTM: long short-term memory.

^h^AE: autoencoder.

^i^HC: handcrafted.

^j^SVM: support vector machine.

^k^CNN: convoluted neural network.

^l^EF: early fusion.

**Table 2:** Multiple modalities: comparison with other methods.

Study	Value, mean (SD)	Modalities
Werner et al [17]	65.02 (8.72)	Video, ECG^a^, EMG^b^, and EDA^c^
Lopez-Martinez and Picard [36]	66.68 (10.87)	ECG and EDA
Wang et al [37]	70.4 (9.76)	ECG, EMG, and EDA
Subramaniam and Dass [38]	92.604 (3.49)	ECG and EDA
Our method	81.08 (4.59)	EDA, EMG, EDA, and RR^d^

^a^ECG: electrocardiogram.

^b^EMG: electromyogram.

^c^EDA: electrodermal activity.

^d^RR: respiration rate.

## Discussion

### Principal Findings

This study demonstrated that RR emerged as the strongest single-modality predictor of pain intensity, particularly for distinguishing between baseline and lower pain levels. EMG performed best for higher pain intensities, while EDA and ECG showed comparatively lower effectiveness as stand-alone modalities. Multimodal models, though offering potential advantages in robustness and complementary information, generally underperformed compared with the RR single-modality models, likely due to challenges related to noise and data alignment. The study highlights the importance of modality selection and data fusion strategies for pain recognition in postoperative settings.

#### Performance by Modality

##### Pain Recognition Using RR Alone

From the single-modality results (Table 1), it is evident that RR models outperform all other modalities, especially for the BL versus PL1 and BL versus PL2 models. This highlights the strong predictive power of RR in distinguishing between baseline and lower pain intensities. The best-performing model used RR alone. One justification for these results could be the dynamic nature of RR signals in response to pain stimuli. Since we effectively isolated and captured periods of higher pain intensity with smaller window sizes, this could have helped the models better distinguish between baseline and other pain levels.

##### Pain Recognition Using EMG Alone

For the highest pain category (BL vs PL3), the EMG model outperformed other single-modality models. This suggests that facial muscle activation captured by EMG signals is particularly effective for distinguishing higher pain intensities. The comparatively lower performance of other modalities, such as EDA, could be attributed to the subtle variations in their responses to different pain levels.

##### Pain Recognition Using EDA Alone

EDA models exhibited comparatively lower performance across all pain categories. This may be due to the difficulty in capturing clear variations in EDA signal responses to different pain levels, as observed in our experiments.

##### Pain Recognition Using ECG Alone

While ECG features contributed strongly to the performance of multimodal models, their single-modality results were not as robust as those of RR or EMG. However, the best-performing multimodal models shared identical configurations with the best ECG models, suggesting that ECG features had a significant influence on the multimodal results.

#### Challenges With Extremes in Pain Levels

The BL versus PL1 and BL versus PL3 models had relatively poor performances across both single and multimodal approaches. BL versus PL1 struggled to distinguish the baseline from the lowest pain intensity due to the subtlety of the physiological responses collected while experiencing this pain level. The BL versus PL3 model, however, found it challenging to distinguish pain levels due to the scarcity of labels for the highest pain intensity. Although data augmentation can help mitigate these challenges, there is no substitute for real data. On the contrary, the BL versus PL2 models performed better due to the relative abundance of such labels reported during trials.

#### Multimodal Performance

The best-performing multimodal model was trained on automatic features outputted from an LSTM network with a 10-second window size. This model, which made use of strong labels without any data augmentation techniques, achieved comparable results to the best-performing ECG single-modality model. Early fusion outperformed late fusion, likely due to its ability to detect correlations across modalities during feature selection [39]. By treating each modality as independent, late fusion might lose correlations in the combined feature space.

However, single-modality models, particularly RR, generally outperformed multimodal models. This contrasts with previous studies on healthy participants, where multimodal approaches typically excelled. Our findings suggest that the unique challenges of real-world postoperative data, including noise and missing signals, may complicate the integration of multiple modalities.

#### Advantages and Trade-Offs

While multimodal models have the potential to add complementary information and robustness, they also introduce challenges related to data alignment and noise management. Single-modality models, by contrast, are simpler, easier to interpret, and computationally less expensive. These advantages make single modalities, such as RR and EMG, attractive for certain applications despite the overall potential of multimodal approaches. Multiple modalities certainly have the potential to add more useful information over a single modality and can be used to introduce complementary information and resiliency when any one modality fails or is too noisy [40].

While comparing our results to previous studies [17,36-38] in Table 2, it can be observed that our models outperform most of their models in mean pain assessment scores except Subramaniam and Dass [38]. However, this is not entirely a fair comparison because we use 3 pain levels instead of 4, and our patients are not healthy.

An additional consideration is the comfort and compliance of patients wearing multiple biosensors, especially in postoperative settings. While multimodal models rely on multiple sources of data, this could pose a burden to patients who may already be experiencing discomfort. Future iterations of the framework could focus on optimizing the number of biosensors by identifying the most informative modalities. This optimization could improve patient compliance while maintaining the accuracy and robustness of the system.

#### Limitations

The main limitation of our algorithm is the presence of noise in the form of motion artifacts produced while collecting physiological signals. Since we obtained data from real postoperative patients in a clinical setting, they were allowed to move more freely compared to experiments performed in controlled laboratory settings. The presence of these motion artifacts diminished the quality of our data, thus negatively impacting our machine learning algorithms.

In addition, our study was conducted in a setting with a limited and relatively homogeneous patient population. While this setting allowed us to focus on developing and testing the algorithm, it restricts the generalizability of our findings to broader and more diverse clinical environments. Testing the model in varied clinical settings and across a larger, more diverse patient population is essential for evaluating its scalability and effectiveness in real-world scenarios. This remains an important future research direction.

Furthermore, we must acknowledge the more complicated facets of pain that are not fully captured by our algorithm, such as the number of days after surgery, the amount of pain medication administered, and the location and type of pain experienced. Incorporating these factors in future studies could improve the accuracy and robustness of pain assessment systems.

### Future Directions

One of the main research directions we would like to explore is the development of real-time multimodal pain assessment systems using deep learning architectures. In such scenarios, missing or incomplete data from one or more modalities are likely to be encountered. Real-time systems also face limitations related to computational complexity and power constraints. Building on the experiments conducted in this study, we aim to create models capable of dynamically determining which modalities to use in an energy-efficient manner without compromising performance given the clinical context.

In addition, a promising avenue for future work is to build personalized machine learning models. These models could leverage data from groups of similar patients while being fine-tuned to make predictions for individual patients. This personalized approach accounts for the large interindividual variability in pain perception, which makes a monolithic model unsuitable. Previous research has demonstrated the feasibility of using multitask machine learning to address variability in mood prediction tasks [41]. This strategy could be extended to the domain of pain assessment, not only for acute postoperative pain but also for chronic pain scenarios. Personalized modeling will be a vital step toward creating clinically viable and effective pain assessment algorithms.

### Conclusions

In this paper, we presented a multimodal machine learning framework for classifying pain in real postoperative patients using the iHurt Pain Database. Both traditional handcrafted features and deep learning–generated automatic features were extracted from physiological signals (ECG, EDA, EMG, and PPG). Several experiments were conducted to perform binary classification among 3 different pain intensities versus baseline levels of pain. Models were varied based on the modalities used, the data augmentation techniques applied (SMOTE, Snorkel, or both), the machine learning algorithms used, and the modality fusion methods implemented.

Our results showed that binary pain classification significantly benefits from the application of data augmentation techniques in conjunction with automatic features. The single-modality models based on RR and EMG outperformed the multimodal models. The BL versus PL3 model with the best results was trained on EMG data alone, highlighting the importance of facial muscle activation in distinguishing higher pain intensities from baseline levels. This finding is consistent from a clinical perspective, as higher pain intensities are commonly associated with acute pain.

Overall, this study highlights a novel approach to addressing the challenges of building a pain recognition system for real postoperative patients, particularly constraints such as label imbalances and missing data. By employing robust data preprocessing techniques, data augmentation strategies, and multimodal fusion approaches, our framework demonstrates the potential for accurate and objective pain classification in clinical settings. These findings lay the groundwork for advancing multimodal pain assessment methods tailored to real-world clinical scenarios.

## Abbreviations

AdaBoost: adaptive boosting
AE: autoencoder
CNN: convolutional neural network
CPOT: Critical Care Pain Observation Tool
ECG: electrocardiogram
EDA: electrodermal activity
EMG: electromyogram
KNN: k-nearest neighbors
LSTM: long short-term memory
NRS: Numerical Rating Scale
PPG: photoplethysmography
RF: random forest
RR: respiration rate
SMOTE: synthetic minority oversampling technique
SVM: support vector machine
